# Proton-pump inhibitors are associated with a reduced risk for bleeding and perforated gastroduodenal ulcers attributable to non-steroidal anti-inflammatory drugs: a nested case-control study

**DOI:** 10.1186/ar2207

**Published:** 2007-05-23

**Authors:** Harald E Vonkeman, Robert W Fernandes, Job van der Palen, Eric N van Roon, Mart AFJ van de Laar

**Affiliations:** 1 Department of Rheumatology and Clinical Immunology, Medisch Spectrum Twente Hospital and University of Twente, Ariensplein 1, 7500 KA, Enschede, The Netherlands; 2 Stroinkslanden Pharmacy, Veldhoflanden 90, 7542 LX, Enschede, The Netherlands; 3 Department of Clinical Epidemiology and Statistics, Medisch Spectrum Twente Hospital, Haaksbergerstraat 55, 7500 KA, Enschede, The Netherlands; 4 Department of Clinical Pharmacy and Clinical Pharmacology, Medisch Centrum Leeuwarden, Henri Dunantweg 2, 8934 AD, Leeuwarden, The Netherlands

## Abstract

Treatment with non-steroidal anti-inflammatory drugs (NSAIDs) is hampered by gastrointestinal ulcer complications, such as ulcer bleeding and perforation. The efficacy of proton-pump inhibitors in the primary prevention of ulcer complications arising from the use of NSAIDs remains unproven. Selective cyclooxygenase-2 (COX-2) inhibitors reduce the risk for ulcer complications, but not completely in high-risk patients. This study determines which patients are especially at risk for NSAID ulcer complications and investigates the effectiveness of different preventive strategies in daily clinical practice. With the use of a nested case-control design, a large cohort of NSAID users was followed for 26 months. Cases were patients with NSAID ulcer complications necessitating hospitalisation; matched controls were selected from the remaining cohort of NSAID users who did not have NSAID ulcer complications. During the observational period, 104 incident cases were identified from a cohort of 51,903 NSAID users with 10,402 patient years of NSAID exposure (incidence 1% per year of NSAID use, age at diagnosis 70.4 ± 16.7 years (mean ± SD), 55.8% women), and 284 matched controls. Cases were characterised by serious, especially cardiovascular, co-morbidity. In-hospital mortality associated with NSAID ulcer complications was 10.6% (incidence 21.2 per 100,000 NSAID users). Concomitant proton-pump inhibitors (but not selective COX-2 inhibitors) were associated with a reduced risk for NSAID ulcer complications (the adjusted odds ratio 0.33; 95% confidence interval 0.17 to 0.67; *p *= 0.002). Especially at risk for NSAID ulcer complications are elderly patients with cardiovascular co-morbidity. Proton-pump inhibitors are associated with a reduced risk for NSAID ulcer complications.

## Introduction

Treatment with non-steroidal anti-inflammatory drugs (NSAIDs) is known to be complicated by gastrointestinal toxicity. NSAIDs impair prostaglandin-dependent gastric mucosal protective mechanisms. When these defences have been breached, a second wave of injury caused by luminal gastric acid may facilitate deeper ulceration [1]. Prevention of gastroduodenal ulcers attributable to the use of NSAIDs may target the inhibition of gastric acid secretion with histamine-2 receptor antagonists (H2RAs) or proton-pump inhibitors (PPIs). Alternatively, locally depleted endogenous cytoprotective prostaglandins may be replaced by the administration of prostaglandin E_1 _analogues, such as misoprostol. Several studies have evaluated and compared these strategies [2]. High-dose misoprostol is effective in the primary prevention of endoscopic NSAID ulcers and also NSAID ulcer complications, such as bleeding and perforation, but is often poorly tolerated because of diarrhoea and abdominal discomfort [3]. Elevation of the intragastric pH by PPIs and high-dose H2RAs reduces the risk of endoscopic NSAID ulcers [2]. In direct comparison, PPIs show an efficacy comparable to that of misoprostol, but they are better tolerated [4]. Furthermore, PPIs are more effective in the prevention of NSAID ulcers than low-dose H2RAs [5]. However, the efficacy of PPIs and H2RAs in the primary prevention of clinically relevant endpoints, such as bleeding and perforated NSAID ulcers, remains unproven.

The discovery of the isoenzymes cyclooxygenase (COX)-1 and COX-2 made it possible to develop highly selective COX-2 inhibitors [6]. The hypothesis is that COX-1 is expressed constitutively and regulates normal physiology, such as the maintenance of gastric mucosal integrity. Conversely, COX-2 is expressed selectively after exposure to inflammatory mediators or trauma, and has a role in inflammation and pain [7]. In randomised controlled clinical trials, selective COX-2 inhibitors have demonstrated a decreased risk for NSAID ulcers and also ulcer complications [8-11]. Furthermore, in elderly patients with a recent history of bleeding NSAID ulcers, secondary prevention (preventing recurrent bleeding) with a selective COX-2 inhibitor seems comparable to combining a non-selective NSAID with a PPI, although in that study the number of cases was small and neither strategy provided adequate protection [12].

Because of their relatively low incidence, severe gastrointestinal ulcer complications such as bleeding and perforated ulcers can be evaluated most effectively in large observational studies [13]. Randomised controlled clinical trials are designed to evaluate the efficacy of a certain strategy, and despite including thousands of patients they may fail to detect infrequent or long-term complications or side effects. Furthermore, rigorous inclusion and exclusion criteria are maintained, and those at high risk for drug side effects or complications are usually excluded. Conversely, in daily clinical practice, it is especially at-risk patients who are likely to be treated with these new strategies under the assumption of safe, evidence-based pharmacotherapy. Although observational studies are subject to possible bias, they best reflect daily clinical practice and are well suited to study infrequent and long-term complications and side effects. Therefore, to determine the characteristics of patients who are especially at risk for serious NSAID ulcer complications and to compare the effectiveness of different preventive strategies in daily clinical practice, we conducted a large nested case-control study.

## Materials and methods

This nested case-control study was performed within the government-initiated healthcare region of the city of Enschede in The Netherlands. On 31 December 2003 the population consisted of 152,989 persons living in a well-defined geographically isolated area largely bordering on Germany. All in-patient healthcare is provided by a single teaching hospital, supplied with all diagnostic and therapeutic facilities. All drug prescriptions are registered in electronic prescription records of 14 local pharmacies. Most drugs, including NSAIDs, are provided by the patient's own pharmacy, directly reimbursed by the healthcare system. A cohort of NSAID users can be identified continuously from the electronic prescription records.

Serious NSAID ulcer complications were defined as ulcerations of the stomach or proximal duodenum causing perforation, obstruction or bleeding that occurred during the use of NSAIDs, necessitating hospitalisation of the patient.

### Selection of cases

During a prospective 26-month observational period (November 2001 to December 2003), we identified all consecutive NSAID users who were hospitalised with serious NSAID ulcer complications. Most patients were identified during endoscopy or abdominal surgery. A few patients were identified on the basis of a clinical presentation of upper gastrointestinal bleeding alone, with haematemesis or melaena, if no further diagnostic procedure was performed because of co-morbidity or advanced age. In some of these patients the diagnosis was confirmed during autopsy. Patients were included in the study if they used NSAIDs (including selective COX-2 inhibitors) at the time of diagnosis of a gastroduodenal ulcer. Aspirin in high dosage (more than 100 mg daily) was considered to be a NSAID. As soon as possible after the diagnosis, patients were given a questionnaire on their sociodemographic characteristics, actual and recent medication, co-morbidity and medical history. The questionnaire contained specific items on the use of NSAIDs, aspirin, anticoagulants, gastroprotective drugs, and steroids, and also on the history of gastroduodenal events. For verification of the questionnaires, we reviewed the medical charts of all cases, as well as reports on endoscopy, surgery and pathology. Medication use before and during hospitalisation, as reported by the patient, was verified by reviewing prescription registrations provided by the in-hospital and community-based pharmacies. Patients were interviewed by one of the authors (HV) if ambiguities were encountered in the questionnaires or during verification.

Patients were excluded if they reported not having used NSAIDs, if endoscopy, surgery or autopsy did not reveal gastroduodenal ulcers, if ambiguities remained despite interviewing the patient, if a malignancy of the stomach was diagnosed or if another reason for upper intestinal bleeding (such as esophagogastric varices, arteriovenous malformations, diffuse gastritis or Mallory–Weiss tears) was diagnosed.

### Selection of controls

Matched controls were selected from the remaining cohort of NSAID users. For selecting controls, index dates were defined as the day on which an NSAID ulcer was diagnosed in each of the cases. Controls were frequency-matched on sex and age, and had to be using NSAIDs (including selective COX-2 inhibitors) on the index date. Selected controls were asked to complete the same questionnaire as the cases. Medication use as reported by the controls was verified by reviewing prescription databases. Controls were interviewed if ambiguities were encountered in the questionnaires or during verification. All non-responders were sent a second identical questionnaire. Finally, a random sample of non-responders was telephoned to detect bias in non-responding.

### Statistical analysis

In univariate analyses, potential confounding continuous variables were analysed with Student's *t*-test and nominal data were analysed with Pearson χ^2 ^tests or Fisher's exact tests for small numbers. Multivariate analyses were performed by using logistic regression with NSAID ulcers as the dependent variable. A full model consisting of all significant and other likely causational variables was reduced stepwise to a parsimonious model. All *p *values were two-sided, and *p *≤ 0.05 was regarded as significant. All analyses were performed with SPSS for Windows, version 12.0.1 (SPSS, Chicago, IL, USA).

The study was approved by the Medical Ethics Reviewing Committee of the Medisch Spectrum Twente Hospital. There were no external sources of funding or study sponsors.

## Results

Over the 26-month prospective observational period the cohort of NSAID users contained 51,903 NSAID users with 10,402 patient years of NSAID exposure. From this cohort, 104 cases were hospitalised with serious NSAID ulcer complications. Because of the geographically isolated position, referral to other hospitals, especially for acute gastrointestinal events, is extremely rare. Therefore, in this population the incidence of hospitalisation due to serious NSAID ulcer complications can be reliably calculated at 1% per year of NSAID use.

Table 1 shows demographic characteristics and co-morbidities. The typical case is an elderly patient, age at diagnosis 70.4 ± 16.7 years (mean ± SD; range 22 to 98 years), 55.8% were female. Many patients reported concurrent disease or previous medical events suggesting serious, especially cardiovascular, co-morbidity. This self-reported co-morbidity was supported by the concomitant medication used (Table 2). The 104 cases together used 12 different NSAIDs (Table 2). The duration of NSAID use before the gastrointestinal event varied; the median was 1.13 months (interquartile range 10 days to 12 months). Most patients did not exceed their prescribed maximum daily dose. However, occasional use of more than one NSAID simultaneously was reported by 12 patients (11.5%).

**Table 1:** Sociodemographic characteristics and co-morbidities for cases and controls

Characteristic	Cases (*n *= 104)	Controls (*n *= 284)	OR	95% CI	*p*
Age at diagnosis (years)	70.4 ± 16.7	67.1 ± 14.3			
Female sex	58 (55.8)	163 (57.4)			
Body mass index (kg/m^2^)	24.7 ± 4.7	26.7 ± 4.6	-	-	0.001
Smoking	28 (26.9)	51 (18)	1.96	1.15–3.37	0.01
Alcohol (glasses per week)	9.6 ± 33.2	6.2 ± 8.6	-	-	0.12
Coffee (cups per week)	18.9 ± 20.6	22.8 ± 13.8	-	-	0.06
Education low vocational or less	39 (56.5)	176 (64.0)	0.73	0.43–1.25	0.25
Married	42 (46.7)	166 (59.3)	0.60	0.37–0.97	0.04
Medical history					
Hypertension	30 (28.8)	95 (33.5)	0.81	0.49–1.32	0.39
Heart failure	26 (25.0)	32 (11.3)	2.63	1.48–4.67	0.001
COPD	25 (24.0)	57 (20.1)	1.26	0.74–2.15	0.40
Myocardial infarction	20 (19.2)	32 (11.3)	1.88	1.02–3.45	0.04
Stroke	18 (17.3)	28 (9.9)	1.91	1.01–3.63	0.04
Heart rhythm disturbance	18 (17.3)	52 (18.3)	0.93	0.52–1.69	0.82
Diabetes mellitus	16 (15.4)	33 (11.6)	1.38	0.73–2.64	0.32
Anaemia	16 (15.4)	32 (11.3)	1.43	0.75–2.74	0.28
Renal insufficiency	16 (15.4)	15 (5.3)	3.26	1.55–6.86	0.001
Previous gastrointestinal ulcers	16 (15.4)	33 (11.7)	1.37	0.72–2.60	0.34
Malignancy	15 (14.4)	26 (9.2)	1.67	0.85–3.30	0.14
Rheumatoid disease, including OA	42 (40.4)	97 (34.2)	1.31	0.82–2.07	0.26

Scores are means ± SD or number of patients (percentage). OR, unadjusted odds ratio; CI, confidence interval; COPD, chronic obstructive pulmonary disease; OA, osteoarthritis.

**Table 2:** NSAIDs and concurrent medication in use at the time of the gastrointestinal event

Medication	Cases (*n *= 104)	Controls (*n *= 284)	OR	95% CI	*p*
Non-selective NSAIDs					
Indometacin	3 (2.9)	4 (1.4)	2.08	0.46–9.45	0.39
Naproxen	10 (9.6)	14 (4.9)	2.05	0.88–4.78	0.09
Diclofenac	44 (42.3)	108 (38.0)	1.20	0.76–1.89	0.44
Diclofenac–misoprostol	8 (7.7)	19 (6.7)	1.16	0.49–2.74	0.73
Other NSAIDs	3 (2.9)	8 (2.8)	1.03	0.27–3.94	1.00
Ibuprofen	16 (15.4)	69 (24.3)	0.57	0.31–1.03	0.06
High-dose aspirin (>100 mg/day)	2 (1.9)	0 (0.0)	-	-	0.07
Selective NSAIDs					
Rofecoxib	16 (15.4)	42 (14.8)	1.05	0.56–1.96	0.88
Celecoxib	1 (1.0)	8 (2.8)	0.34	0.04–2.71	0.46
Meloxicam	1 (1.0)	12 (4.2)	0.22	0.03–1.71	0.20
Gastroprotective drugs					
Proton-pump inhibitors	14 (13.5)	77 (27.1)	0.42	0.23–0.78	0.005
H2RAs	4 (3.8)	9 (3.2)	1.22	0.37–4.06	0.74
Misoprostol	8 (7.7)	20 (7.0)	1.10	0.47–2.58	0.83
Additional risk factors					
High-dose NSAID	11 (10.6)	17 (6.0)	1.86	0.84–4.11	0.12
More than one NSAID	12 (11.5)	54 (19.0)	0.56	0.28–1.09	0.08
Low-dose aspirin (≤ 100 mg/day)	32 (30.8)	69 (24.3)	1.39	0.84–2.28	0.20
Clopidogrel/dipyridamole	5 (4.8)	9 (3.2)	1.54	0.51–4.72	0.54
Coumarin	14 (13.5)	19 (6.7)	2.17	1.05–4.51	0.04
Low-molecular-mass heparin	13 (12.5)	2 (0.7)	20.14	4.46–90.94	<0.001
SSRIs	6 (5.8)	9 (3.2)	1.87	0.65–5.39	0.24
Corticosteroids	14 (13.5)	32 (11.3)	1.23	0.63–2.40	0.55
Analgesics					
Acetaminophen	45 (43.3)	54 (19.0)	3.25	1.99–5.29	<0.001
Tramadol	12 (11.5)	6 (2.1)	6.04	2.21–16.56	<0.001
Morphine	6 (5.8)	2 (0.7)	8.63	1.71–43.48	0.006
Cardiovascular drugs					
Diuretics	34 (32.7)	57 (20.1)	1.93	1.17–3.20	0.009
ACE inhibitors	24 (23.1)	32 (11.3)	2.36	1.32–4.25	0.003
Digoxin	8 (7.8)	11 (3.9)	2.09	0.82–5.35	0.12
Beta-blockers	22 (21.2)	64 (22.5)	0.92	0.53–1.59	0.77
Nitrates	8 (7.7)	26 (9.2)	0.83	0.36–1.89	0.65
Calcium-channel blockers	10 (9.6)	35 (12.3)	0.76	0.36–1.59	0.46
Lipid-lowering drugs	9 (8.7)	38 (13.4)	0.61	0.29–1.32	0.21
Oral glucose-lowering drugs	12 (11.5)	15 (5.3)	2.34	1.06–5.18	0.03
Benzodiazepines	34 (32.7)	65 (22.9)	1.64	0.99–2.68	0.05
Inhalator therapy	22 (21.2)	56 (19.7)	1.09	0.63–1.90	0.76
DMARDs	14 (13.5)	20 (7.0)	2.05	0.99–4.23	0.05

Scores are number of patients (percentage). NSAIDs, non-steroidal anti-inflammatory drugs; OR, unadjusted odds ratio; CI, confidence interval; H2RAs, histamine-2 receptor antagonists; SSRIs, selective serotonin re-uptake inhibitors; ACE, angiotensin-converting enzyme; DMARDs, disease-modifying anti-rheumatic drugs. High-dose NSAID is more than the daily defined dose.

In most cases (80 patients, 76.9%), serious NSAID ulcer complication was the reason for presentation and hospitalisation. In the remainder a serious NSAID ulcer complication took place during hospitalisation for another reason. Characteristics of the gastrointestinal events are presented in Table 3. No diagnostic procedure was performed in only six (5.8%) patients, because of co-morbidity or advanced age. The mean haemoglobin level at presentation was 6.1 ± 1.9 mmol/l (mean ± SD; range 1.8 to 9.8). In those using coumarin, the international normalized ratio (INR) at presentation was 4.87 ± 1.41 (mean ± SD) but the mean haemoglobin level at presentation did not differ from that in patients not taking coumarin, and neither did the number of units of blood administered during hospitalisation.

**Table 3:** Characteristics of the gastrointestinal event attributable to use of non-steroidal anti-inflammatory drugs

Characteristic	Number (percentage)
Clinical presentation	
Melaena	65 (62.5)
Haematemesis	28 (26.9)
Perforation	12 (11.5)
Stomach pain	21 (20.2)
Collapse	16 (15.4)
No previous stomach complaints	57 (54.8)
Ulcer location	
Gastric	53 (51.0)
Duodenal	34 (32.7)
Both gastric and duodenal	11 (10.6)
No diagnostic procedure performed	6 (5.7)
Ulcer perforation	14 (13.5)
*Helicobacter pylori*	
Positive	21 (20.2)
Negative	45 (43.3)
Not tested	38 (36.5)

The total number of patients was 104.

Mortality due to serious NSAID ulcer complications was high: 11 patients (10.6%) died in hospital, and another 4 (3.8%) died within 3 months of the diagnosis. The incidence of in-hospital mortality due to serious NSAID ulcer complications can be calculated at 21.2 per 100,000 NSAID users.

For 104 cases, 757 controls were selected from the remaining cohort of NSAID users. On receiving the first questionnaire 225 controls responded, of whom 203 were included. On receiving a second questionnaire, a further 123 responded, of whom 81 were included. From the 64 excluded responders, 18 questionnaires were returned by someone other than the selected control, 15 denied taking NSAIDs, 17 refused, 1 had been hospitalised in a psychiatric hospital, 1 was a case who had already been included as such, and for 12 controls relatives informed us that the selected person had died. In the group of 20 randomly selected non-responders who were telephoned, no bias for non-responding was found.

In total 284 controls, frequency matched for age and sex, with NSAID use on the index date were included. Demographic characteristics, co-morbidities and current medication use are summarised in Tables 1 and 2. The mean age was slightly lower for the controls than for the cases because insufficient numbers of controls could be found for some of the extremely elderly cases.

### Statistical results

In univariate analysis, cases and controls differ significantly with regard to body mass index, smoking habits, marital status, medical history of heart failure, myocardial infarction, stroke and renal insufficiency (Table 1). Significant differences in medication use were found for PPIs, coumarin, low-molecular-mass heparin, analgesics, diuretics, angiotensin-converting-enzyme inhibitors, oral glucose-lowering drugs, benzodiazepines and disease-modifying anti-rheumatic drugs (Table 2).

Concomitant use of PPIs was significantly higher in the controls than in the cases (cases 13.5%; controls 27.1%; *p *= 0.005). Use of selective COX-2 inhibitors was comparable (cases 16.4%; controls 17.6%; *p *= 0.77). Use of the preferential COX-2 inhibitor meloxicam differed, but not significantly, and numbers were small (cases 1%; controls 4.2%; *p *= 0.20).

A full logistic regression model of all significant and other likely causational variables was reduced stepwise to a parsimonious model, finally containing concomitant use of PPIs, low-molecular-mass heparin, acetaminophen, coumarin, and history of heart failure (Table 4). Use of selective COX-2 inhibitors was not associated with a significantly reduced risk for serious NSAID ulcer complications (*p *= 0.74); neither was the use of preferential COX-2 inhibitors (*p *= 0.22). Concomitant use of PPIs was associated with a significantly reduced risk for serious NSAID ulcer complications (adjusted odds ratio 0.33; 95% confidence interval 0.17 to 0.67; *p *= 0.002).

**Table 4:** Multivariate analysis of significant variables and other likely causational variables for serious NSAID ulcer complications

Predictor	Adjusted OR	95% CI	*p*
Proton-pump inhibitors	0.33	0.17–0.67	0.002
Coumarin	2.09	0.93–4.70	0.075
Heart failure	2.44	1.28–4.66	0.007
Acetaminophen	2.80	1.64–4.79	<0.001
Low-molecular-mass heparin	17.33	3.71–80.95	<0.001

Serious non-steroidal anti-inflammatory drug (NSAID) ulcer complication was the dependent variable. Only variables from the final parsimonious model are shown. OR, odds ratio; CI, confidence interval.

In a post hoc subgroup analysis of selective COX-2 inhibitor users, there were no significant differences in concomitant use of low-dose aspirin (8 cases (47%); 19 controls (38%); *p *= 0.51), non-selective NSAIDs (3 cases (18%); 10 controls (20%); *p *= 0.83) or PPIs (3 cases (18%); 17 controls (34%); *p *= 0.20); neither were there significant differences in concomitant use of coumarin, heparin, steroids or high-dose H2RAs or in ulcer history.

Furthermore, among those taking selective COX-2 inhibitors, cases and controls did not differ significantly with regard to the number of risk factors for NSAID-associated gastropathy, suggesting comparable risk profiles. Similarly, in a post hoc subgroup analysis for those taking either proton-pump inhibitors or high-dose H2RAs, cases and controls again did not differ significantly with regard to the number of risk factors for NSAID-associated gastropathy.

In six patients no diagnostic procedure was performed because of co-morbidity or advanced age. In a post hoc analysis these patients with probable NSAID ulcers were compared with the 98 patients with definite NSAID ulcers. Significant differences between patients with probable or definite NSAID ulcers were age (mean 87.3 and 69.4 years, respectively; *p *= 0.01), medical history of diabetes mellitus, chronic obstructive pulmonary disease and in-hospital mortality (66.7% and 7.1%, respectively; *p *= 0.001). Excluding these patients with probable NSAID ulcers from the cases did not significantly change the results of the univariate or multivariate analyses.

In 24 patients, serious NSAID ulcer complications occurred in hospital. These patients were compared with the 80 patients who presented with NSAID ulcer complications. Significant differences between in-hospital or presenting patients were sex (37.5% and 61.3% female, respectively; *p *= 0.04), ulcer history (29.2% and 11.3%, respectively; *p *= 0.03), medical history of a malignancy, diabetes mellitus, use of oral glucose-lowering drugs and use of low-molecular-mass heparin (45.5% and 3.8%, respectively; *p *< 0.001). Exclusion of these in-hospital patients from the cases resulted in a significant change in the univariate analyses for use of oral glucose-lowering drugs (cases 6.3%; controls 5.3%; *p *= 0.74) and for use of low molecular mass heparin (cases 3.8%; controls 0.7%; *p *= 0.04). The results of the multivariate analysis also changed (Table 5).

**Table 5:** Multivariate analysis after exclusion of patients with in-hospital NSAID ulcer complications

Predictor	Adjusted OR	95% CI	*p*
Proton-pump inhibitors	0.31	0.15–0.66	0.002
Coumarin	2.38	1.03–5.48	0.04
Heart failure	2.10	1.04–4.21	0.04
Acetaminophen	2.47	1.39–4.39	0.002
Low-molecular-mass heparin	6.06	0.91–40.60	0.06

Serious non-steroidal anti-inflammatory drug (NSAID) ulcer complication was the dependent variable. Only variables from the final parsimonious model are shown. OR, odds ratio; CI, confidence interval.

## Discussion

In this nested case-control study, the concomitant use of proton-pump inhibitors was associated with a two-thirds reduction in the risk for serious NSAID ulcer complications. The efficacy of PPIs in the primary prevention of NSAID-associated gastropathy has so far only been proven for subjective symptoms and surrogate endpoints, such as dyspepsia and endoscopic ulcers, and in the secondary prevention of serious NSAID ulcer complications, PPIs do not seem to prevent recurrence [12,14,15]. Our data suggest that PPIs may be effective in the primary prevention of clinically relevant bleeding and perforated NSAID ulcers, confirming other recent observational studies [16-18]. However, randomised controlled trials powered on these hard endpoints need to be conducted to prove efficacy.

It is noteworthy that in this study the use of selective COX-2 inhibitors was not associated with protection for serious NSAID ulcer complications. Lack of protection from selective COX-2 inhibitors could not be explained by confounders such as concomitant use of aspirin, coumarin, heparin or steroids or by ulcer history. Previous studies demonstrating the efficacy of selective COX-2 inhibitors in the primary prevention of NSAID ulcer complications largely excluded high-risk patients, whereas in high-risk patients selective COX-2 inhibitors may fail to prevent the recurrence of NSAID ulcer bleeding [12,14,15]. Although neither selective COX-2 inhibitors nor concomitant PPIs seem to be entirely effective in preventing the recurrence of ulcer complications, our data suggest that PPIs may be superior to selective COX-2 inhibitors in the primary prevention of NSAID ulcer complications.

Cases used coumarin more often than controls (adjusted odds ratio 2.09; 95% confidence interval 0.93 to 4.70; *p *= 0.075). Furthermore, in those cases using coumarin, the mean INR at presentation was 4.87 ± 1.41 (mean ± SD) and one-third (5 patients) had an INR greater than 6.5. Although no INR was measured in the controls, it is possible that this elevated INR contributed to these patients developing serious NSAID ulcer bleeding.

Cases used low-molecular-mass heparin significantly more often than controls (adjusted odds ratio 17.33; 95% confidence interval 3.71 to 80.95; *p *< 0.001). In addition, cases used acetaminophen significantly more often than controls (adjusted odds ratio 2.80; 95% confidence interval 1.64 to 4.79; *p *< 0.001). It is possible that these differences reflect in-hospital treatment protocols rather than a true elevated risk for serious NSAID ulcer complications. However, an increased risk for NSAID ulcers with concomitant high-dose acetaminophen has been reported previously [13]. Exclusion of patients with in-hospital NSAID ulcer complications truncated the odds ratio for low-molecular-mass heparin (adjusted odds ratio 6.06; 95% confidence interval 0.91 to 40.60; *p *= 0.06; Table 5).

Cases reported a history of heart failure significantly more often than controls (adjusted odds ratio 2.44; 95% confidence interval 1.28 to 4.66; *p *= 0.007). The association between heart failure and risk for NSAID-associated gastropathy has previously been demonstrated, but a credible causational mechanism remains to be identified [19].

One of the strengths of this study is that it reflects daily clinical practice. The large randomised controlled clinical trials that demonstrated the efficacy and safety of selective COX-2 inhibitors were conducted in relatively young, healthy subjects. Our study suggests that these may not be the patients who are especially at risk for serious NSAID ulcer complications and confirms another recently conducted large nested case-control study that also found no evidence for enhanced gastrointestinal safety with selective COX-2 inhibitors [20]. Another strength of our study lies in the robustness of the data. Gastrointestinal events in cases and controls were verified, as were data on actual medication used. Other groups have studied populations of up to several thousand patients, but associations were derived by coupling databases and the validity of the data was not always verified [21,22].

The local infrastructure makes it unlikely that many cases were missed. However, one weakness of this study is that underestimation of the number of events might still have occurred. Another weakness of this study, as in any case-control study, is the possibility of selection bias. Although we have controlled for all known possible confounders, selection by indication or an unknown confounding mechanism cannot be excluded with certainty.

## Conclusion

Serious NSAID ulcer complications have a significant mortality rate: 10.6% die in hospital and 14.4% within 3 months of the event. At risk are especially elderly patients with cardiovascular co-morbidity. In daily clinical practice, the concomitant use of PPIs is associated with a two-thirds reduction in the risk for serious NSAID ulcer complications.

## Abbreviations

COX = cyclooxygenase; H2RAs = histamine-2 receptor antagonists; INR = international normalized ratio; NSAIDs = non-steroidal anti-inflammatory drugs; PPIs = proton-pump inhibitors.

## Competing interests

The authors declare that they have no competing interests.

## Authors' contributions

All authors contributed significantly to writing the article. HEV and RWF also contributed to the selection and inclusion of cases and controls. JvdP also contributed to the statistical analysis of the data. All authors read and approved the final manuscript.
